# High-throughput sequencing to evaluate the effects of methamphetamine on the succession of the bacterial community to estimate the postmortem interval

**DOI:** 10.1080/20961790.2022.2046368

**Published:** 2022-05-31

**Authors:** Shujuan Wang, Wei Chen, Yanjie Shang, Lipin Ren, Xiangyan Zhang, Yadong Guo, Changquan Zhang

**Affiliations:** Department of Forensic Science, School of Basic Medical Sciences, Central South University, Changsha, China

**Keywords:** Forensic sciences, methamphetamine, microbes, bacterial community succession, postmortem interval, high-throughput sequencing

## Abstract

In forensic medical examinations, estimating the postmortem interval (PMI) is an important factor. Methamphetamine (MA) is a synthetic stimulant that is commonly abused, and estimation of the PMI after MA abuse has become one of the main tasks in forensic investigation. Microorganisms play a vital role in carrion decomposition. Analysing the bacterial succession patterns can be used as a forensic tool to estimate the PMI. The present study aimed to analyse bacterial succession changes during the decomposition of MA to estimate the PMI. We analysed bacterial communities in rabbits treated with three different concentrations of MA (0, 22.5, and 90 mg/kg) under the natural conditions of 20 °C and 70% humidity by sequencing 16S rRNA gene amplicons using the Illumina MiSeq system. We obtained 2 374 209 high-quality sequences and 2 937 operational taxonomic units (OTUs). The relative abundances of the bacterial communities varied markedly in response to different MA concentrations. Interestingly, in response to the different concentrations of MA, Bacteroidetes became disparate in the rectum in the late PMI. Increased numbers of bacterial taxa were identified in the rectum and buccal cavity samples, except at the highest concentration of MA in the rectum samples when PMI was 0–h, than were present in live rabbits. Meanwhile, the PMI correlated significantly with bacterial succession at different taxonomic levels. Our results suggested that bacterial community succession could be used as a “microbial clock” to estimate the PMI in cases of MA-related death; however, further study is required to gain a deeper understanding of this concept.

## Key points

As a forensic tool, microbiome succession patterns have great potential for PMI estimation.

At different taxonomic levels, the variation in the succession of microorganisms under three different concentrations of MA during the processes of decomposition is significantly related to PMI for estimating MA abuse.

These findings provide a new idea for the estimation of PMI of MA abused by changes in microorganisms.

## Introduction

Estimation of the postmortem interval (PMI) is an important and difficult aspect of forensic investigation. Generally, the PMI refers to the period from when death occurs to when the corpse is found, which can also be called the “postmortem experience time” [1,2]. Currently, most methods to estimate PMI rely on the morphological method, including early postmortem phenomena (algor mortis, rigor mortis, and livor mortis) and late postmortem phenomena (greenish discolouration, putrefactive blister, and putrefactive networks) [3–5]. However, decomposition is a complex process, which is greatly affected by biological (cellular enzymes, microbes, and insects) and environmental (temperature, weather, and humidity) factors [6], and can only produce a rough estimate of the PMI. In addition, other methods have been used to estimate the PMI, such as the developmental or evolutionary patterns of necrophilic insects [7–12] and genomic and transcriptomic methods [13–16]. However, necrophilic insect activities are affected by sunlight, temperature, and weather, which might lead to inaccurate estimates of the PMI [17]. Similarly, genomics and transcriptomics are affected by multiple circumstantial and environmental factors, in which the accuracy and precision decrease as the PMI increases. Therefore, despite decades of research, the accuracy of estimating the PMI has not improved significantly, and no method can be used reliably to estimate the PMI accurately [18]. To overcome the limitations in practice, the great potential of the microbiome as a tool for estimating the PMI in forensic has emerged.

Microorganisms are a form of ubiquitous evidence that present regardless of the season, which often responds to changes in their environment in a predictable way. The postmortem microbial community’s response to PMI is predicable [19–21]. Specifically, Metcalf et al. [22] reported the use of a mouse model that allowed them to propose the concept of a “microbial clock”, indicating that the microbial community decomposition of cadavers might be developed as a forensic tool to assess the PMI. Similarly, Pechal et al. [20] used a swine carcasses model to reveal a significant negative linear relationship between the microbial phyla and the richness of family taxa during the decomposition progresses. In summary, these studies indicated that the succession of microorganisms during the decomposition processes seems to be a foreseeable process, which has a significant impact on estimating the PMI in forensic practice. Previous studies have shown that during PMI estimation, drugs and poisons could change the direction of microorganisms succession [23–25]. Therefore, when estimating the PMI *via* changes in the microbiome, it is necessary to consider the effects of drugs or poisons on the succession of microbial communities.

Methamphetamine (MA) is a synthetic stimulant that is commonly abused in many countries worldwide. Generally, the phenomenon of MA abuse has become a global problem. With the prevalence of MA abuse, negative socioeconomic, cognitive, and legal consequences have followed [26]. Therefore, in cases of suicides or accidents, estimation of the PMI of MA abuse has become one of the main tasks in forensic investigation. In recent years, studies have shown that MA could change the development time of the larvae of necrophilic insects and cause deviations in the estimated PMI [5,27,28]. Among these, MA can change the balance of intestinal microbes. Ning et al. [29] proved that the use of MA caused changes in the intestinal flora of rats in a study of MA-induced conditioned place preference rats. Chen et al. [30] revealed that MA promotes intestinal inflammation by increasing the relative abundance of pathogenic bacteria in the intestine and reducing the expression of tight junction (TJ) proteins in the intestines. Although studies have shown that the abuse of MA can lead to significant time- and structure-dependent changes in the diversity of the gut microbiome and taxonomic structure, only a handful of studies have described the relationship between the effect of MA on the microbiome and PMI estimation.

In the present study, we aimed to explore the relationship between the changes in the buccal cavity and rectum microbiome for estimating the PMI under three different concentrations of MA using high-throughput sequencing. The results revealed that at different taxonomic levels, the succession of microorganisms is related significantly to the PMI for estimating the use of MA.

## Materials and methods

### Study sites and grouping of samples

As models of human decomposition, we used adult female New Zealand rabbit carcasses to study the changes in the bacterial community during decomposition under three different concentrations of MA. The experiments were conducted in December, 2019 in Changsha City, Hunan Province, Central South China. The laboratory temperature was approximately 20 °C with 70% humidity and natural light. The rabbits, each weighing 2.0 kg, were divided randomly into three groups. In the control group (Group A), 0.6 mL saline solution was injected through the ear margin veins, and death was caused by air embolization through the ear margin veins at 1.5 h after injection. Two different doses (22.5 and 90 mg/kg) of MA, diluted in 0.6 mL of saline solution, were injected into the ear margin veins. MA at 22.5 mg/kg represented the half lethal concentration (Group B), and in this group, death was caused by air embolization through the ear margin veins at 1.5 h after injection. MA at 90 mg/kg represented twice the lethal concentration (Group C) and caused immediate death. Each corpse was individually put into plastic boxes and placed into the centre of the laboratory. The boxes contained sliver sand to absorb the putrefactive liquid to avoid contamination of the surroundings. To simulate human decomposition, the necrophagous insects had access to the corpses in the natural environment. Each day, the physical changes in the carcasses were observed and the surface changes were recorded.

### Collecting samples of the bacterial community

We sampled the bacterial communities before death (5 min before killing), immediately after death (less than 10 min postmortem), and at 2 h, 6 h, 12 h, 24 h, 3 d, 5 d, 10 d, and 20 d postmortem. Sterile cotton applicators scrubbed with sterile ultrapure water were used to swab the rectum and buccal cavity lightly for 1 min. The cotton applicator was transferred to a 1.5 mL sterile Eppendorf tube with 1 mL sterile ultrapure water. Samples were then snap frozen at −80 °C for later use.

### Extraction of DNA, PCR amplification, and sequencing

Total bacterial DNA was extracted from the New Zealand rabbit buccal cavity and rectum samples using a MoBio PowerSoil DNA Isolation Kit (Mo Bio Laboratories, Carlsbad, CA, USA), following the manufacturer’s instructions. The concentration and purity of the DNA were assessed using 1% agarose gel electrophoresis. In 1.5 mL sterile Eppendorf tubes, the DNA samples were diluted using sterile water to 1 ng/mL. In each group, the DNA samples from the rabbit carcasses were mixed in equal concentrations at each time point. Novogene Biological Information Technology Co. (Beijing, China) completed the sequencing operation using Illumina MiSeq sequencing.

A 515F/806R primer set 50 was used to conduct the PCR reactions [31], amplifying the 16S rRNA gene V3–V4 hypervariable regions (forward primer: 5′-GTGCCAGCMGCCGCGGTAA-3′, reverse primer: 5′-GGACTACHVGGGTWTCTAAT-3^′^). The reverse primer included a 6-bp error-correcting barcode that was unique for each sample. The PCR reactions comprised 15 μL of Phusion High-Fidelity PCR Master Mix (New England Biolabs, Ipswich, MA, USA), 0.2 μmol/L forward and reverse primers, and 10 ng of template DNA in a 30-μL reaction volume. The PCR reactions were carried out as follows: 98 °C for 1 min; 30 cycles of 98 °C for 10 s, 50 °C for 30 s, and 72 °C for 30 s; a final 5 min extension at 72 °C. Agarose gel electrophoresis (2%) was used to confirm the resulting amplicons. A GeneJET Gel Extraction Kit (Thermo Fisher Scientific, Carlsbad, CA, USA) was used to purify the 400–450 bp amplicons. The amplicons were quantified, and equal amounts of the purified amplicons were combined into a single tube. An NEB Next Ultra DNA Library Prep Kit for Illumina (New England Biolabs) was used to generate the sequencing libraries, according to the suppliers protocols, including the addition of index codes. A Qubit 2.0 Fluorometer (Thermo Fisher Scientific) and an Agilent Bioanalyzer 2100 system (Agilent, Santa Clara, CA, USA) were used to assess the quality of the libraries. The Illumina MiSeq platform (Illumina, San Diego, CA, USA) was used for sequencing, generating 300 bp paired-end reads.

FLASH version 1.2.7 was used to merge the paired-end reads from the original DNA fragments [32]. Low-quality parts were removed using QIIME version 1.7.0, and the primer sequences and barcodes were trimmed produce the original reads [33]. QIIME was used to filter out sequences ambiguous bases (N) or low-quality bases [34] and UCHIME was used to remove chimeric sequences to obtain clean reads [35]. The unique barcodes on the remaining sequences were used to assign them to the samples, and before statistical analysis, the sequences were subjected to rarefaction at the level of 9200.

The UPARSE pipeline (version 7.0.1001) was used to cluster the sequences [36], and a threshold of 97% similarity was used to assign similar sequences to operational taxonomic units (OTUs). Among the similar sequences, a representative sequence from each OTU was identified, which was the longest sequence with the maximum number of hits to the other representative sequences. Using a minimum identity of 80%, the representative sequences were aligned at the GreenGenes database [37]. PyNAST software (version 1.2) was used to determine the phylogenetic relationships among the representative sequences, and for rapid multiple sequence alignment [31], and the GreenGenes database “Core Set” data [38] were used. “Rare taxa” were defined as those taxa with a relative abundance <1% of the total abundance of all samples. Unclassified reads were categorised as “others”.

Three phylogenetic diversity metrics were used to assess the α-diversity: the Shannon index, Chao 1, and Observed species. According to Observed species, we generated rarefaction curves. Both unweighted and weighted UniFrac distances were used to evaluate the β-diversity between the bacterial communities [39]. The unweighted pair group method with arithmetic mean (UPGMA) was used to stratify the hierarchical clustering of the samples. The UniFrac distances of weighted and unweighted distance matrices were subjected to principal components analysis (PCoA) to visualise the difference in bacterial community structure and composition, respectively. Based on the Bray-Curtis dissimilarity distance matrices, analysis of similarities description was performed to examine the differences in the community composition between the groups of samples [40].

## Results

The study was performed at a daily temperature of approximately 20 °C with 70% humidity, in a reversed light cycle of 12 h:12 h light:dark. The decomposition process of the control and treatment groups showed no obvious differences according to the physical changes of the carcasses. In the current study, we observed five stages of decomposition according to the physical changes: fresh, bloated, active decay, advanced decay, and dry remains. In the first stage of fresh decomposition (0–1 d), the necrophagous insects arrived at the carrion immediately, indicating that the presence of MA in the rabbits did not impede their activities. Within the bloated stage of death (2–3 d), necrophagous insect eggs were oviposited on eyes and noses of the cadavers. During the active decomposition stage (4–5 d), a strong odour emerged and putrefactive fluid leakage was observed. The characteristics of the advanced decomposition stage (6–10 d) were the lack of odour, the removal of most of the soft tissues, and a high number of necrophagous insect larvae. Decomposition finished with the dry remains (11–20 d) and during this stage, we observed post-feeding larvae and pupa of the necrophagous insects.

**Figure 1.**
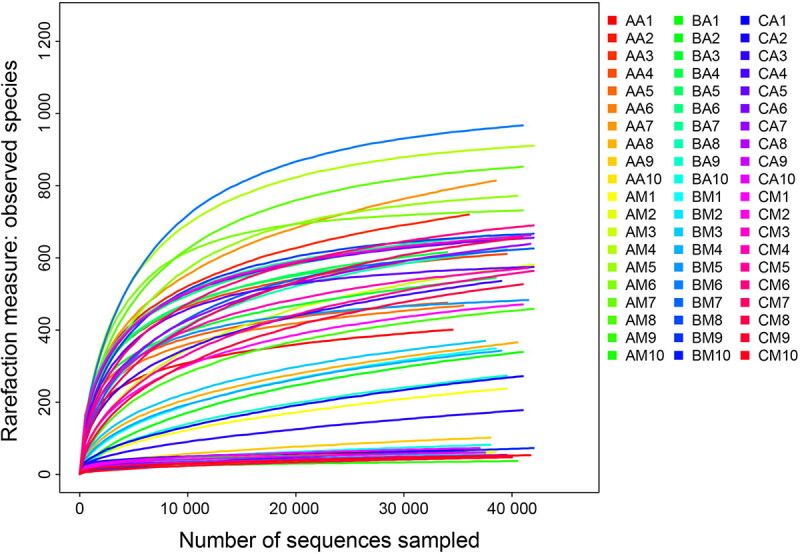
Rarefaction curves of observed species number clustered at 97% sequence identity across all samples. Sample named to refer to samples as described in Table 1.

Thirty buccal cavity samples and 30 rectum samples were collected at three different MA concentrations (0, 22.5, and 90 mg/kg). Sequencing was successful for all sample. After quality filtering, failed sample and low numbers of sequences removal, 2 545 378 raw sequences were generated, among which we retained 2 374 209 high-quality sequences with an average length of 252 bases. For each sample, the mean number of sequences was 4 242. The species representation in each sample had approached the plateau phase according to the rarefaction curves, thus additional sequencing effort was unlikely to detect more bacteria (Figure 1). The UPARSE pipeline clustered the high-quality sequences into 2 937 OTUs at a 97% identity threshold.

**Figure 2.**
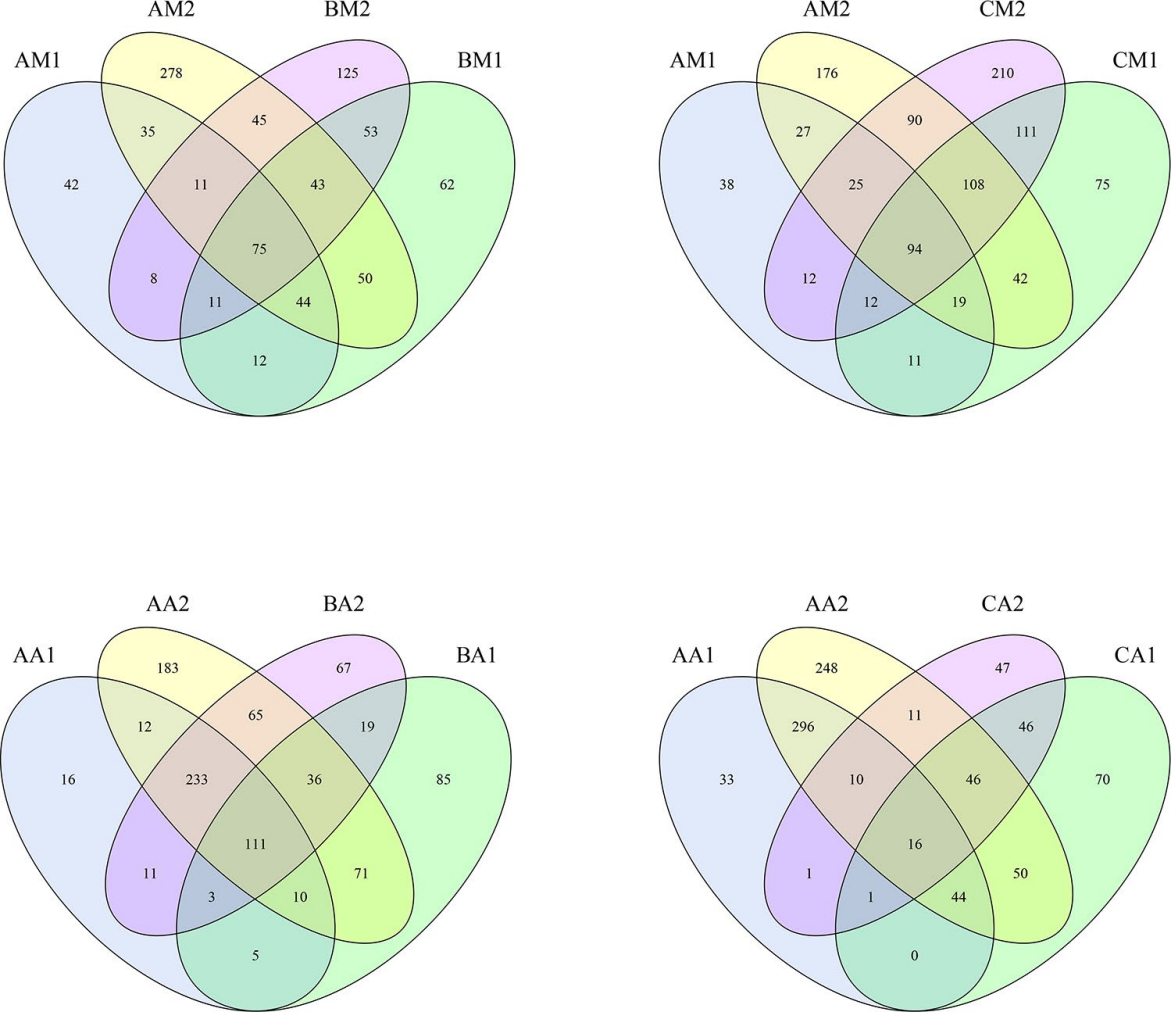
Shared operational taxonomic unit (OTU) analysis of the different communities. Venn diagrams showing the unique and shared OTUs in the different communities, (A) for the AM1, AM2, BM1, and BM2 communities, (B) for the AM1, AM2, CM1, and CM2 communities, (C) for the AA1, AA2, BA1, and BA2 communities and (D) for the AA1, AA2, CA1, and CA2 communities. Sample names refer to samples as described in Table 1.

The sequencing results identified bacteria from 34 phyla, 27 classes, 42 orders, 65 families, 54 genera, and 47 species. Table 1 shows the detailed characteristics of each sample. Except for Group C, compared with the live rabbit-derived samples, the OTU number and α-diversity metrics for the rectal and buccal cavity samples increased rapidly immediately after death (Table 1). The similarities and differences between the communities in the different samples were compared using a Venn diagram (Figure 2). The AM1, AM2, BM1, and BM2 communities shared 75 common OTUs (Figure 2A). The AM1, AM2, CM1, and CM2 communities shared 94 common OTUs (Figure 2B). The BM1, BM2, CM1, and CM2 communities shared 116 common OTUs (Supplementary Figure 1A). The AA1, AA2, BA1, and BA2 communities shared 111 common OTUs (Figure 2C). The AA1, AM2, CA1, and CA2 communities shared 16 common OTUs (Figure 2D). The BA1, BA2, CA1, and CA2 communities shared 36 common OTUs (Supplementary Figure 1B).

**Table 1.** Operational taxonomic unit (OTU)-based diversity index in New Zealand rabbit buccal cavity and rectum samples during decomposition.

Sample name	Sample group	Sample region	Sample time	Raw sequences	Clean sequences	OTUs	Chao 1	Observed species	Shannon index
AM1	Group A	Buccal cavity	Alive	45 348	42 485	238	359.33	238	1.87
AM2	Group A	Buccal cavity	0 h	47 843	42 986	581	705.32	581	2.23
AM3	Group A	Buccal cavity	2 h	48 713	43 012	911	946.25	911	4.58
AM4	Group A	Buccal cavity	6 h	45 512	42 229	772	801.38	772	4.20
AM5	Group A	Buccal cavity	12 h	45 878	42 114	732	758.00	732	4.57
AM6	Group A	Buccal cavity	1 d	47 970	42 852	853	890.68	853	4.21
AM7	Group A	Buccal cavity	3 d	46 569	42 943	460	526.41	460	1.71
AM8	Group A	Buccal cavity	5 d	43 887	42 161	37	56.50	37	1.13
AM9	Group A	Buccal cavity	10 d	44 334	42 127	56	73.50	56	1.72
AM10	Group A	Buccal cavity	20 d	45 575	42 512	60	79.43	60	1.39
AA1	Group a	Rectum	Alive	50 581	42 793	401	478.03	401	4.30
AA2	Group a	Rectum	0 h	49 489	42 829	721	900.15	721	4.93
AA3	Group a	Rectum	2 h	46 426	42 859	612	671.35	612	4.71
AA4	Group a	Rectum	6 h	47 917	43 259	476	542.07	476	4.44
AA5	Group a	Rectum	12 h	48 195	42 497	468	561.72	468	4.29
AA6	Group a	Rectum	1 d	48 865	43 086	816	1 034.48	816	4.76
AA7	Group a	Rectum	3 d	45 781	42 614	366	556.12	366	2.28
AA8	Group a	Rectum	5 d	44 537	42 221	102	154.56	102	2.04
AA9	Group a	Rectum	10 d	45 778	42 867	62	108.00	62	1.62
AA10	Group a	Rectum	20 d	47 138	42 792	65	98.00	65	2.18
BM1	Group B	Buccal cavity	Alive	44 923	41 923	350	521.27	350	2.37
BM2	Group B	Buccal cavity	0 h	42 889	41 643	371	496.26	371	2.85
BM3	Group B	Buccal cavity	2 h	43 256	41 950	343	460.16	343	2.34
BM4	Group B	Buccal cavity	6 h	45 642	42 150	483	534.00	483	4.25
BM5	Group B	Buccal cavity	12 h	46 444	42 228	968	1 007.51	968	4.66
BM6	Group B	Buccal cavity	1 d	49 463	43 118	626	665.07	626	3.66
BM7	Group B	Buccal cavity	3 d	47 913	43 042	667	685.42	667	4.15
BM8	Group B	Buccal cavity	5 d	44 666	42 697	73	125.93	73	0.69
BM9	Group B	Buccal cavity	10 d	45 979	42 684	51	70.43	51	1.49
BM10	Group B	Buccal cavity	20 d	46 415	42 636	57	74.14	57	1.80
BA1	Group b	Rectum	Alive	46 540	42 297	340	471.09	340	1.56
BA2	Group b	Rectum	0 h	50 243	43 295	545	686.57	545	4.79
BA3	Group b	Rectum	2 h	45 965	42 532	616	677.32	616	4.75
BA4	Group b	Rectum	6 h	47 512	43 027	601	659.45	601	4.84
BA5	Group b	Rectum	12 h	46 253	42 398	662	735.99	662	4.65
BA6	Group b	Rectum	1 d	47 091	42 471	664	784.38	664	4.68
BA7	Group b	Rectum	3 d	46 183	42 297	625	753.89	625	3.15
BA8	Group b	Rectum	5 d	44 888	42 711	275	476.92	275	1.36
BA9	Group b	Rectum	10 d	45 258	42 648	82	148.11	82	2.19
BA10	Group b	Rectum	20 d	45 882	42 344	58	64.88	58	1.75
CM1	Group C	Buccal cavity	Alive	45 712	42 580	472	539.05	472	2.56
CM2	Group C	Buccal cavity	0 h	47 423	42 469	662	729.16	662	4.53
CM3	Group C	Buccal cavity	2 h	45 988	42 303	572	631.21	572	3.66
CM4	Group C	Buccal cavity	6 h	46 393	42 785	564	694.28	564	2.80
CM5	Group C	Buccal cavity	12 h	47 108	43 055	690	755.25	690	3.81
CM6	Group C	Buccal cavity	1 d	47 373	42 798	657	733.42	657	3.46
CM7	Group C	Buccal cavity	3 d	45 692	42 224	528	624.23	528	2.22
CM8	Group C	Buccal cavity	5 d	44 076	42 228	47	75.88	47	0.51
CM9	Group C	Buccal cavity	10 d	44 998	42 362	53	76.1	53	0.38
CM10	Group C	Buccal cavity	20 d	44 576	42 121	61	124.33	61	0.98
CA1	Group c	Rectum	Alive	48 480	43 454	273	392.05	273	1.94
CA2	Group c	Rectum	0 h	48 281	42 932	178	318.58	178	1.69
CA3	Group c	Rectum	2 h	47 957	42 881	538	709.59	538	2.92
CA4	Group c	Rectum	6 h	47 662	43 016	575	596.00	575	4.37
CA5	Group c	Rectum	12 h	48 953	42 967	656	694.04	656	4.51
CA6	Group c	Rectum	1 d	47 750	42 977	639	764.56	639	3.25
CA7	Group c	Rectum	3 d	46 471	42 754	54	111.75	54	1.54
CA8	Group c	Rectum	5 d	44 987	42 846	49	73.00	49	1.60
CA9	Group c	Rectum	10 d	44 944	42 377	73	115.86	73	2.11
CA10	Group c	Rectum	20 d	45 718	42 777	68	83.11	68	2.15

**Figure 3.**
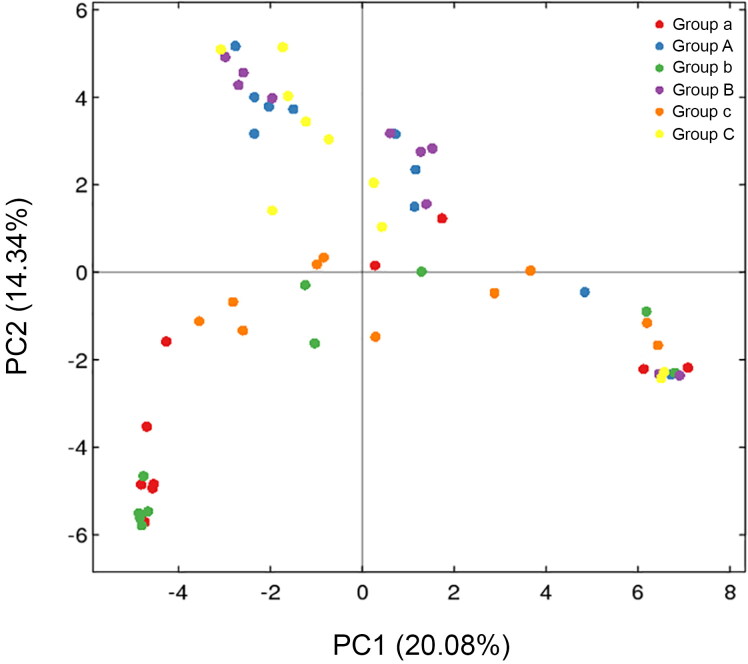
Two-dimensional principal coordinates analysis (PCoA) plot of unweighted UniFrac distance matrices for buccal cavity and rectum samples during decomposition. The bacterial community of the buccal cavity from New Zealand rabbit carcasses in Group A (blue dot), in Group B (purple dot) and Group C (yellow dot), and the bacterial community of the rectum from New Zealand rabbit carcasses in the Group a (red dot), in Group b (green dot) and Group c (orange dot) were represented. Sample names refer to samples as described in Table 1.

**Figure 4.**
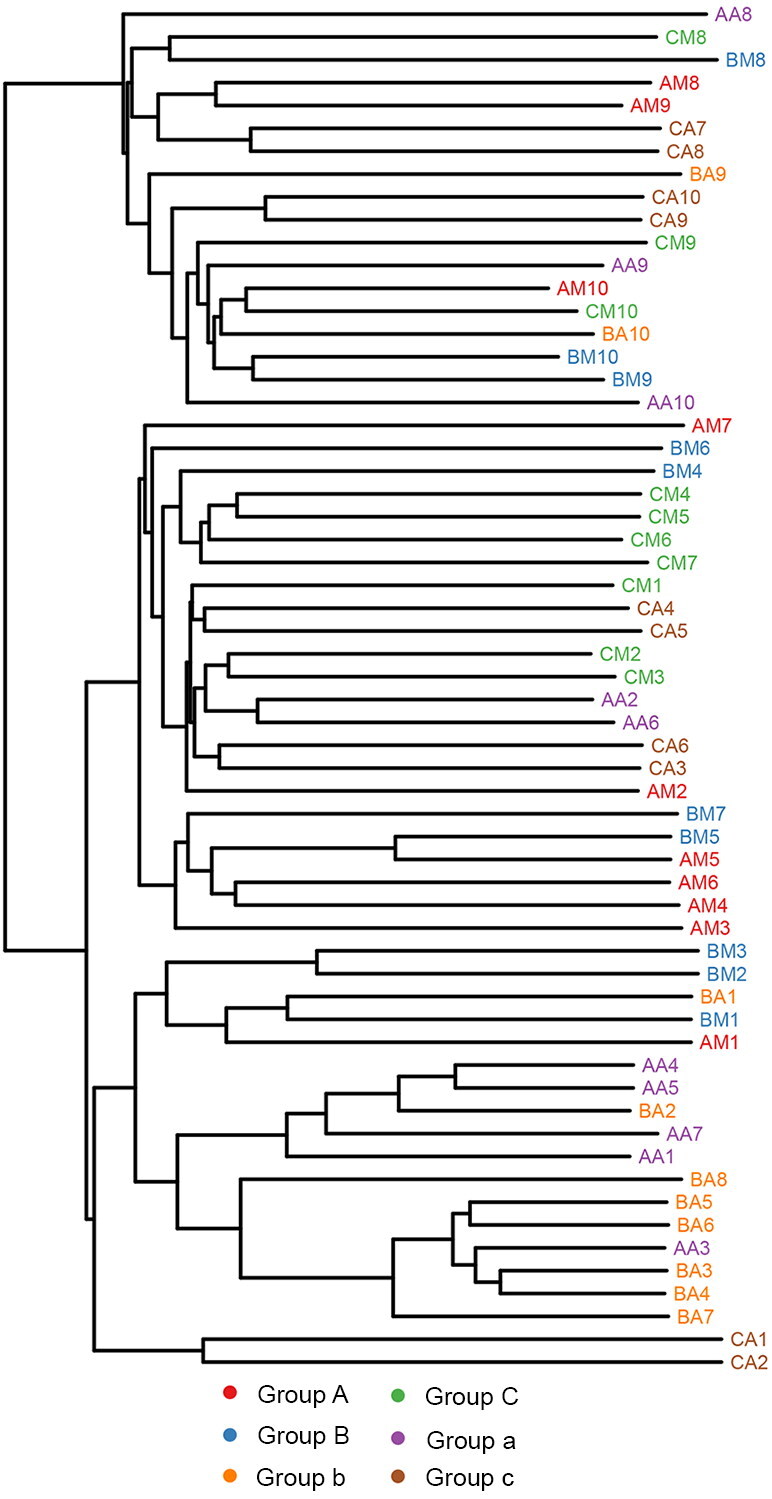
Hierarchical clustering of buccal cavity and rectum samples by unweighted pair group method with arithmetic mean (UPGMA) according to their weighted UniFrac matrix. Sample names refer to samples as described in Table 1.

Principal coordinate analysis (PCoA) of the clustering patterns of samples from the rectum and buccal cavity during decomposition provided the decomposition pattern in a two-dimensional space for subsequent weighted UniFrac distance analysis (Figure 3). The weighted UniFrac PCoA analysis showed that rectal samples collected from rabbits up to 1 d formed a unique cluster that was separated from the samples from the buccal cavity. Principal coordinate 1 (PC1) and Principal coordinate 2 (PC2) analysis (explaining 20.08% and 14.34% of the variance, respectively) indicated that the rectal and buccal microbial communities were separated during decomposition. The hierarchical clustering of buccal cavity and rectum samples using UPGMA permitted analysis of the samples according to their weighted UniFrac matrix. Rectal samples collected from rabbits until 1 d formed a unique cluster that was separated from the samples from the buccal cavity. This result was similar to that of the weighted UniFrac PCoA. Previous studies have demonstrated that the buccal cavity community differs from that of the rectum of cadavers during the expansion phase of decay [41]. In the present study, the buccal cavity and rectal microbial communities were distinguished during decomposition, suggesting that the sequencing data were reliable (Figure 4).

**Figure 5.**
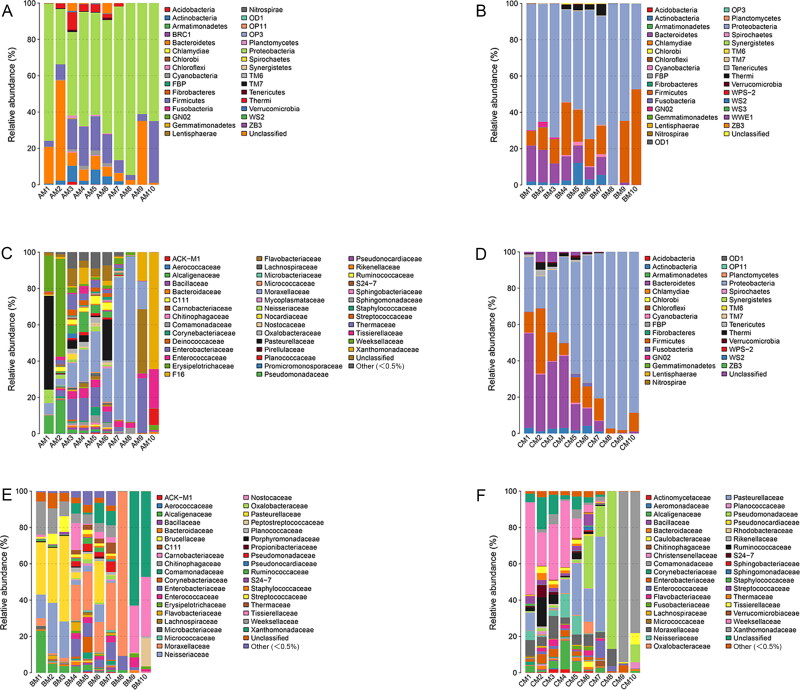
Bacterial community structure variation during decomposition in the buccal cavity at the phylum level and the family level. Relative abundance of bacterial phyla during decomposition in the buccal cavity in Group A (A), the buccal cavity in Group B (B), and buccal cavity in Group C (C). Relative abundance of bacterial families during decomposition in the buccal cavity in Group A (D), the buccal cavity in Group B (E) and buccal cavity in Group C (F). Sample names refer to samples as described in Table 1.

**Figure 6.**
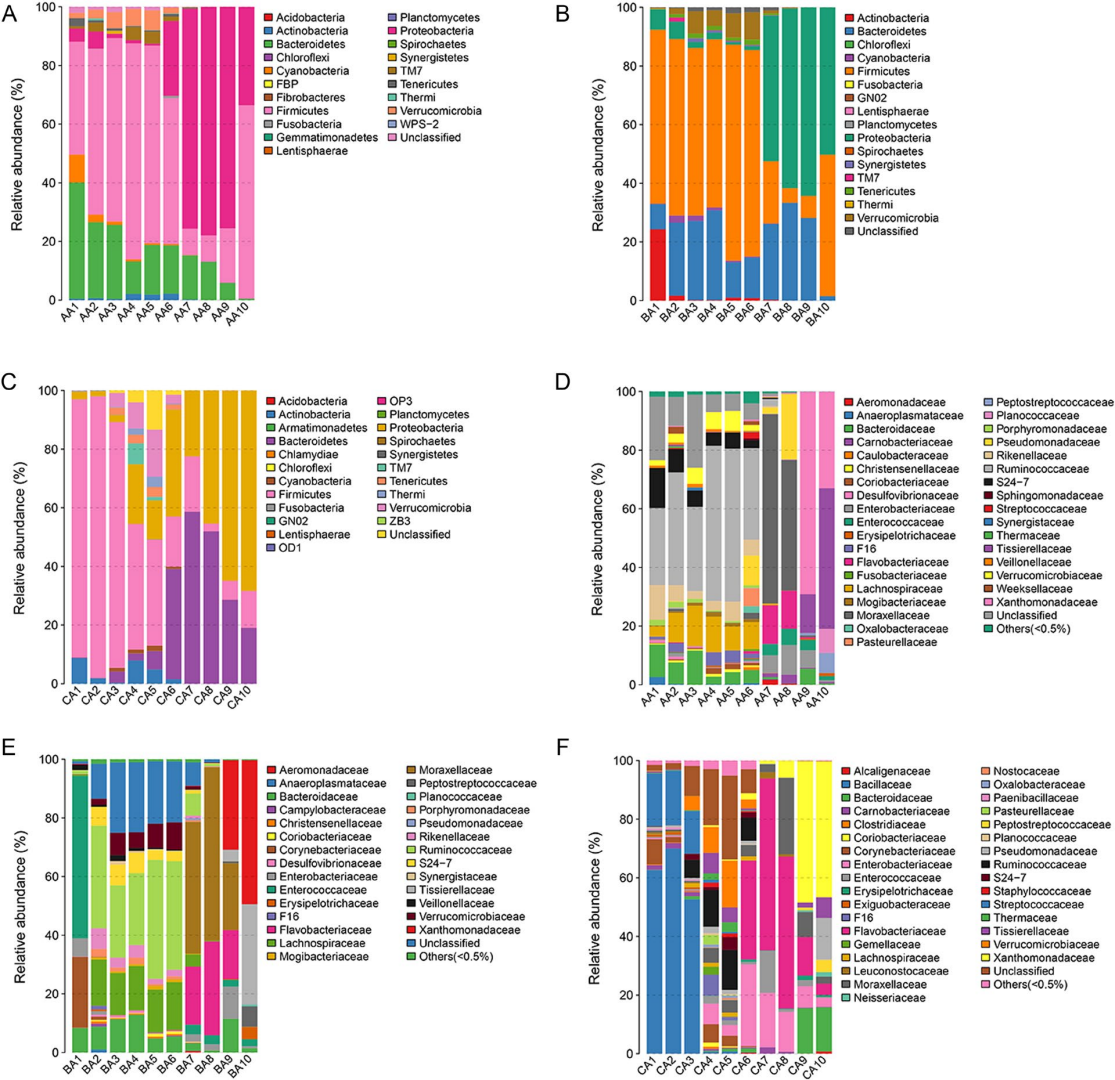
Bacterial community structure variation during decomposition in the rectum at the phylum level and the family level. Relative abundance of bacterial phyla during decomposition in the rectum in Group a (A), rectum in Group b (B), and rectum in Group c (C). Relative abundance of bacterial families during decomposition in the rectum in the Group a (D), the buccal cavity in Group b (E), and the buccal cavity in Group c (F). Sample names refer to samples as described in Table 1.

The 16S RNA sequences were then classified at the phylum and the family levels to identify the succession of the bacterial community structure during decomposition. The relative abundance of different microflora showed obvious changes during the decomposition of the buccal cavity and rectum of rabbit carcasses (Figures 5 and 6). In the buccal cavity (Figure 5A–C), the dominant phylum was the Proteobacteria (63.80% ± 18.32% (SD) in Group A, 65.44% ± 14.92% in Group B, and 62.38 % ± 29.04% in Group C) with the progression of decomposition, except in the immediately after death sample of Group A, in which the Bacteroidetes were the dominant taxon (55.10%). Similarly, there was a large difference between the buccal cavity samples of the experimental group and the control group. At 6 h postmortem, the Proteobacteria occupied the dominant position in Group A (62.73%) and Group B (50.69%), but not in Group C (47.12%). It may be that MA damaged Proteobacteria as the time of death increased. The Bacteroidetes, with a downward trend, were present throughout the decomposition process. From 5 d to 20 d postmortem, the Bacteroidetes increased at first and then decreased in Group A (2.68%, 35.10%, and 1.01%, respectively) and Group B (0.56%, 1.34%, and 0.01%, respectively), while they showed a tendency to increase in Group C (0.07%, 0.37%, and 1.06%, respectively). The rabbit’s corpse is an energy body for microorganisms, however, as corruption progresses, the body’s energy gradually decreases. These data showed that Bacteroidetes were more affected by MA, and the higher the concentration, the stronger the inhibitory effect on the Bacteroidetes. Interestingly, the numbers of Actinobacteria decreased gradually with the progression of decomposition, and almost vanished from 5 d postmortem onwards in all groups. At the family level in the buccal cavity (Figure 5D–F), as decomposition progressed, the numbers of Xanthomonadaceae increased gradually, becoming the dominant taxon at 10 d postmortem in Group B (63.02%) and Group C (93.93%), but not in Group A (15.74%). Different concentrations of MA had marked effects on the Xanthomonadaceae. The results indicated that a lower MA concentration was beneficial for the Xanthomonadaceae, while a high concentration of MA disadvantaged the Xanthomonadaceae.

In all rectum groups (Figure 6A–C), the Bacteroidetes, Firmicutes, and Proteobacteria were the dominant phyla during decomposition. However, they had different successions as the decomposition process progressed. In Group a (0 mg/kg), Firmicutes occupied the dominant position until 1 d postmortem, while the Proteobacteria showed an increasing tendency and became the most abundant phylum, before decreasing from 3 d postmortem to 10 d postmortem. At 20 d postmortem, the Firmicutes displayed a dramatic increase and took over from Proteobacteria as the dominant phylum (65.98%). The Bacteroidetes displayed a tendency of constant decrease during decomposition. In Group b (22.5 mg/kg of MA), the Firmicutes occupied the dominant position until 1 d postmortem, while the Proteobacteria showed an increasing tendency and became the most abundant phylum from 3 d postmortem to 20 d postmortem. At 20 d postmortem, the Firmicutes displayed a dramatic increase and became the second most dominant phylum (48.34%). The Bacteroidetes tended to increase initially and then decrease. In Group c (90 mg/kg of MA), the Firmicutes occupied the dominant position until 12 h postmortem, after which the Bacteroidetes showed an increasing tendency and became the most abundant phylum from 1 d postmortem to 5 d postmortem. At 10 d postmortem to 20 d postmortem, the Proteobacteria displayed a dramatic increase and took over from the Bacteroidetes as the predominant phylum (64.81% and 68.32%, respectively). The above results showed that MA had a more obvious effect on the Firmicutes and Bacteroides. The higher the concentration of MA, the shorter the time that the Firmicutes were the dominant bacteria. Compared with Group a, MA promoted the abundance of the Bacteroides. At the family level in the rectum sample (Figure 6D–F), the Moraxellaceae increased at first and then decreased. At 3 d and 5 d postmortem, the Moraxellaceae occupied the dominant position as the most abundant taxon in Group a and Group b, but not in Group c, in which the Flavobacteriaceae was the predominant family. At 20 d postmortem, the most abundant family in Group a was the Tissierellaceae, while the most abundant family in Group b and Group c was the Xanthomonadaceae.

The difference between the samples treated with MA at 22.5 mg/kg and 90 mg/kg were tested using analysis of similarities description. Between the two groups, we noted differences for several taxa. In the buccal cavity, Capnocytophaga until 1 d postmortem and Elizabethkingia in the late PMI had higher prevalences in Group B than in Group C. Indeed, Capnocytophaga and Elizabethkingia were not detected in Group c (Supplementary Figure 2A–C). In the rectum, Carnobacterium had a higher prevalence in Group b than in Group c, and Anaerococcus was only detected in Group c (Supplementary Figure 2D–F).

## Discussion

In this study of the postmortem microbiome in response to MA, in the buccal cavity of live rabbits, the dominant phyla were Proteobacteria, Bacteroidetes, Firmicutes, and Actinobacteria, and the dominant genera were Actinobacillus and Riemerella. In the rectum of live rabbits, the dominant phyla were Bacteroidetes, Firmicutes, and Proteobacteria, and the dominant genera were Bacteroides and Corynebacterium. We chose the buccal cavity and rectum as the sampling point for microbes because of the convenience of the sampling process, which simplified subsequent analysis. The dominant phyla and genera in the live rabbits were similar to those in samples from the mouth and stool of healthy humans, according to the Human Microbiome Project (HMP) [42]. The HMP reported that the microbiome of the human stool is rich, whereas that of the oral cavity is more limited [42–44], which was similar to our results from samples collected from live rabbits.

New Zealand rabbits not only have similar decay processes to humans, but also harbour similar microbial communities. Among this, similar to research using rat models, the use of rabbit models permitted replicate experiments incorporating a large sample size to be performed. This not only minimized any possible experimental error, but also allowed the assessment of the extent of intra-individual microbiota variation in microbiota that occurred during decomposition [3,6], which indicating that these rabbits are suitable models to study human decomposition.

Compared with that from live rabbits, more bacterial taxa were identified in the buccal cavity immediately after death. The mouth contacts the outside environment directly, and thus external factors, such as humidity and temperature, can easily effect changes in the microbiome. Therefore, to reduce the effects of the external environment to a minimum, we controlled the temperature, humidity, and other controllable factors. In particular, in the rectal samples, there were marked differences pre- and immediately postmortem. Previous studies found no obvious differences in samples collected pre- and immediately postmortem from Sprague Dawley rats [3]. This apparent discrepancy might be caused by species differences between the experimental animals; however, the effect of different concentrations of MA on the experimental animals cannot be ruled out.

According to previous studies, in the bloat stage of decomposition and in healthy humans, the detected oral microbial communities were derived from the gastrointestinal tract communities [41,42]. However, interestingly, we noted that samples from the buccal cavity and rectum were separated in the early PMI, but were clustered together in the late stage. Previous experimental data revealed that after death, saliva, which generates antibacterial activity, ceases to be produced and the intestinal microenvironment can change, thus the limiting factors of microorganisms are gradually weakened [45,46]. Furthermore, with increasing PMI, the differences between the buccal cavity and rectum microorganisms gradually decreased. During the decomposition process, the abundance of bacteria increased, but their richness decreased, which could be used for PMI estimation. In addition, both the rectal and buccal cavity samples contained reads from unclassified organisms that were within acceptable limits. Previous studies revealed that in metagenomic sequencing, approximately 80% of the identified bacteria communities were nonculturable [44]. Although some of the unclassified sequences might represent PCR errors or sequencing artifacts, the large numbers of unclassified sequences indicated the presence of novel bacterial taxa. The presence of novel species can be inferred when the phenotypical characteristics and/or 16S rRNA sequences of unknown bacteria (greater than 0.5% difference) are significantly different from those of the most closely related bacteria. Besides, the bacteria detected on and in a cadaver can be influenced by many factors. For example, the individual’s initial microbiome, the environment surrounding the decomposing cadaver, the method of sample collection, and even variations in swab pressure and the number of swabs used during sample collection.

Our findings revealed that during decomposition, the relative numbers of dominant phyla, i.e. Proteobacteria, Bacteroidetes, Firmicutes, and Actinobacteria, varied significantly, which might assist the estimation of the PMI in cases of MA abuse. At 3 d after death, the Bacteroidetes were prevalent members of the bacterial community in the buccal cavity, becoming even more abundant compared with their level in the control groups (Group A and Group B). Interestingly, the most prevalent phylum on day 10 and day 20 postmortem was the Proteobacteria, which becoming more abundant compared with their levels in the control groups (Group A and Group B). Meat spoilage is commonly associated with the Proteobacteria, and these bacteria have been detected in the skin of slaughtered animals [47]. MA exposure increased the content of conditionally pathogenic bacteria with pro-inflammatory effects, such as the Proteobacteria [30,48–50]. Analysis of the groups with different concentrations of MA revealed that the abundance of the Firmicutes increased during decomposition. The two main phyla in the human gut are reported to be the Bacteroidetes and Firmicutes. In addition, our determination of the relationship between MA administration and intestinal flora revealed that the Firmicutes accounted for the majority of the changed taxa and the propionate-producing genus Phascolarctobacterium was repressed by MA administration [29]. These findings indicated that MA was closely related to changes in the intestinal microbiome. Actinobacteria are widely distributed in terrestrial and aquatic ecosystems, especially in soil, where they play a significant role in the recycling, decomposition, and the formation of refractory biomaterial [51]. Our results further indicated that the Gammaproteobacteria class was predominant in all groups in the rectum and buccal cavity during the late stage of decomposition. Gammaproteobacteria have different metabolic capabilities and can breakdown more complex molecules [52]. Our findings supported the hypothesis that the notable variation of microbial communities might aid PMI estimation in the presence of MA abuse.

## Conclusion

In summary, we sought to explore the relationship between the buccal cavity and rectum microbial changes and the PMI under three different concentrations of MA using high-throughput sequencing technology.

Our results showed that MA induced significant changes in bacterial community succession by decreasing the abundance of probiotics and increasing the abundance of conditioned pathogens with pro-inflammatory effects. As expected, the results support the theory that significant changes in the microbial community might contribute to the estimation of the PMI in the context of MA abuse. Taken together, although further investigation is needed, these findings provide new ideas for the estimation of the PMI in the context of MA abuse using changes in the microbiome. Our research had some limitations. Particularly, despite verifying the effect of MA on the microbial community, we have not thoroughly studied the underlying mechanism of the link between the change of the microbial community caused by MA and the estimation of the PMI. Further experiments are expected to clarify the potential connection between MA-induced microbial community changes and PMI estimation.

## Supplementary Material

Supplemental Material
