# Supplementary figures and images for: High-throughput sequencing to evaluate the effects of methamphetamine on the succession of the bacterial community to estimate the postmortem interval
Source: Forensic Sci Res. 2022 May 31;7(4):736–47. doi: 10.1080/20961790.2022.2046368 (PMC9930777; doi:10.1080/20961790.2022.2046368)

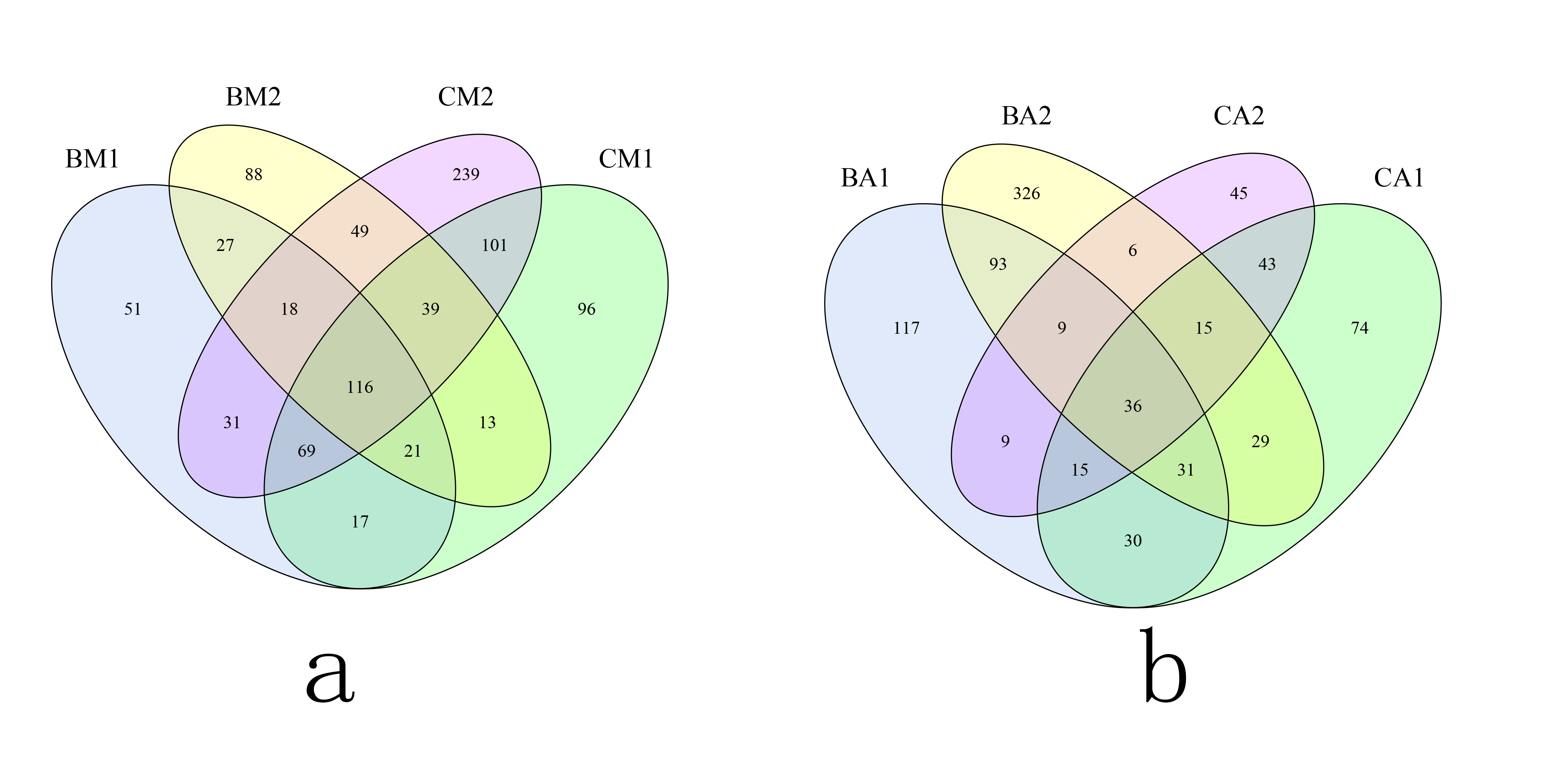

Supplement: Supplemental Material [file TFSR_A_2046368_SM2247.zip › Figure_S1.jpg]

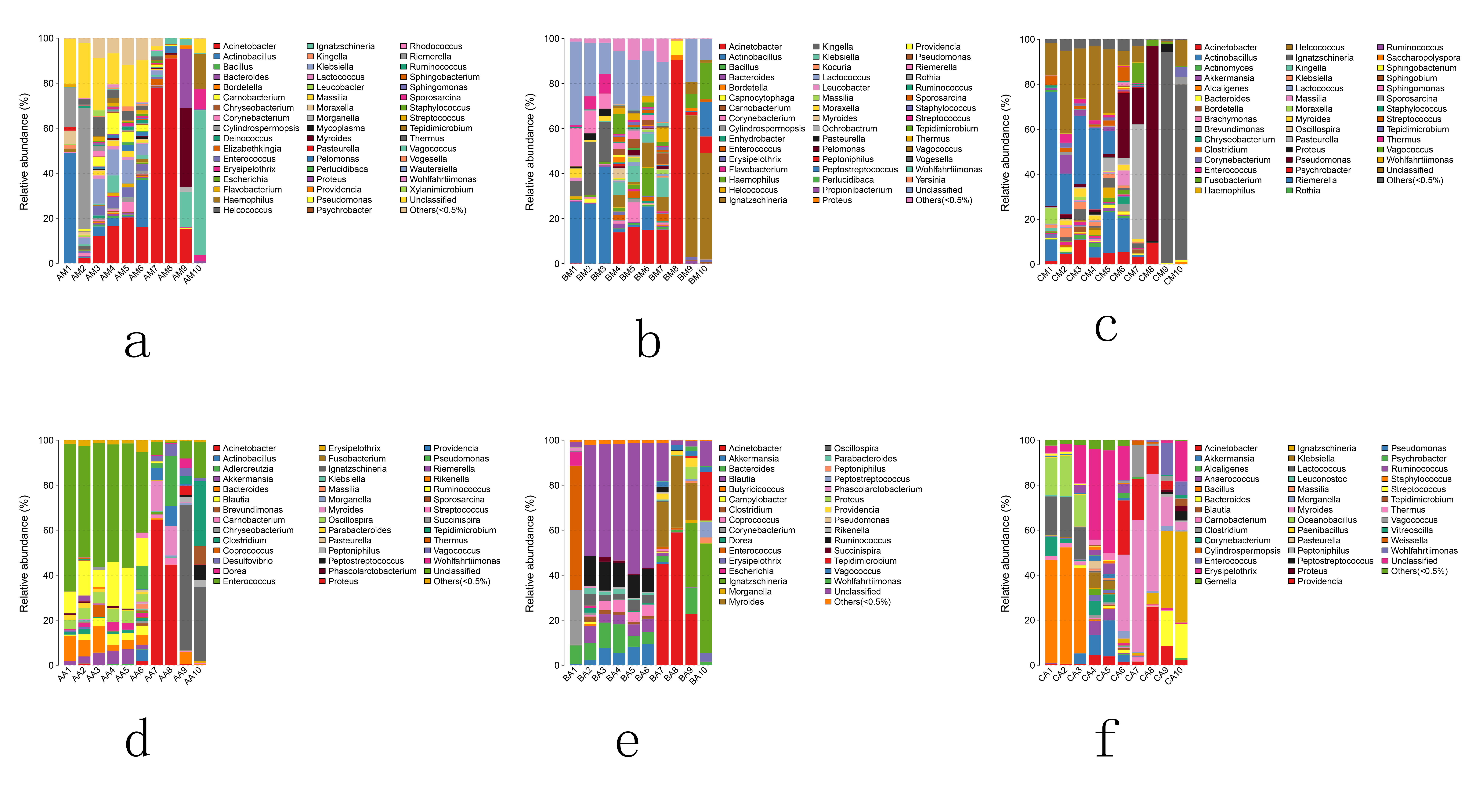

Supplement: Supplemental Material [file TFSR_A_2046368_SM2247.zip › Figure_S2.jpg]
